# Discovery of two novel laccase-like multicopper oxidases from *Pleurotus citrinopileatus* and their application in phenolic oligomer synthesis

**DOI:** 10.1186/s13068-021-01937-7

**Published:** 2021-04-01

**Authors:** A. Zerva, C. Pentari, A. Termentzi, A. H. P. America, D. Zouraris, S. K. Bhattacharya, A. Karantonis, G. I. Zervakis, E. Topakas

**Affiliations:** 1 Industrial Biotechnology & Biocatalysis Group, Biotechnology Laboratory, School of Chemical Engineering, National Technical University of Athens, 5 Iroon Polytechniou Str, Zografou Campus, Athens, Greece; 2 Department of Ophthalmology/Bascom Palmer Eye Institute, University of Miami, Miami, FL 33136 USA; 3 Wageningen Plant Research, Wageningen University and Research, Wageningen, The Netherlands; 4 Laboratory of Physical Chemistry and Applied Electrochemistry, School of Chemical Engineering, National Technical University of Athens, Zografou, Athens, Greece; 5 Laboratory of General and Agricultural Microbiology, Agricultural University of Athens, Athens, Greece

**Keywords:** Laccase-like multicopper oxidases, Biocatalysis, Phenol oligomers

## Abstract

**Background:**

Laccases and laccase-like multicopper oxidases (LMCOs) oxidize a vast array of phenolic compounds and amines, releasing water as a byproduct. Their low substrate specificity is responsible for their tremendous biotechnological interest, since they have been used for numerous applications. However, the laccases characterized so far correspond to only a small fraction of the laccase genes identified in fungal genomes. Therefore, the knowledge regarding the biochemistry and physiological role of minor laccase-like isoforms is still limited.

**Results:**

In the present work, we describe the isolation, purification and characterization of two novel LMCOs, *Pc*Lac1 and *Pc*Lac2, from *Pleurotus citrinopileatus*. Both LMCOs were purified with ion-exchange chromatographic methods. *Pc*Lac2 was found to oxidize a broader substrate range than *Pc*Lac1, but both LMCOs showed similar formal potentials, lower than those reported previously for laccases from white-rot fungi. Proteomic analysis of both proteins revealed their similarity with other well-characterized laccases from *Pleurotus* strains. Both LMCOs were applied to the oxidation of ferulic and sinapic acid, yielding oligomers with possible antioxidant activity.

**Conclusions:**

Overall, the findings of the present work can offer new insights regarding the biochemistry and variability of low-redox potential laccases of fungal origin. Low-redox potential biocatalysts could offer higher substrate selectivity than their high-redox counterparts, and thus, they could be of applied value in the field of biocatalysis.

**Supplementary Information:**

The online version contains supplementary material available at 10.1186/s13068-021-01937-7.

## Background

Multicopper oxidases (MCOs) constitute a group of proteins with a distinctive structure, occupying at least four copper atoms in three different copper centers T1, T2, and T3. Their catalytic mechanism consists of substrate oxidation at a mononuclear copper center (T1) and subsequent electron transfer via a His–Cys–His tripeptide motif to a trinuclear copper center (T1/T2) [1, 2]. Laccases (benzenediol: oxygen oxidoreductases, Lac, EC 1.10.3.2), one of the major MCO superfamilies, act on phenolic substrates, by catalyzing the one-electron oxidation of phenolic hydroxyl groups to phenoxy radicals, reducing O_2_ to water by a four-electron transfer [3]. They are highly oxidizing enzymes, with E^0′^ values varying between + 500 and + 800 mV vs NHE (normal hydrogen electrode) [4]. Hence, their substrate spectrum includes mono-, di-, polyphenols, methoxy-substituted phenols, aromatic compounds, and amines, such as acrylamines or aminophenols. A study upon the catalytic efficiency of four laccases from basidiomycetes revealed that in the case of monophenolic substrates, E^0′^ values of the enzyme play a defining role in substrate transformation, whereas for larger substrates, the structure of the active site is of equal importance [5]. Their biological functions involve lignin biosynthesis and degradation, morphogenesis, stress response, pigment and melanin formation, plant pathogenesis, and insect sclerotization [2, 3].

An interesting characteristic of basidiomycetes is the presence of multiple copies of laccase genes in their genome, also known as laccase multigene families. Such multigene families usually contain four to eight genes and encode a variety of isoenzymes. A typical fungal multigene family has been found to encode up to 17 laccase isoenzymes in *Coprinopsis cinerea* [6], while a greater variety of laccase isoforms is attributed to post-translational modifications. Moreover, laccases from different strains of the same species can possess distinct properties. Glazunova et al. [7] highlighted the importance of glycosylation at modulating catalytic properties of four laccases from different strains of *Steccherinum ochraceum*. However, isoenzymes can differ considerably regarding their biochemical characteristics and can also have different roles in the physiology of the microorganism [8]. A typical example of laccase multigene families is the existence of 12 phenol oxidase (laccase) genes in *Pleurotus ostreatus* [9]. Laccase gene transcription is found to be regulated by culture conditions, while enzymatic activities and gene transcription profiles differ even between closely related strains [9]. Additionally, Park et al. [10] revealed that the expression of those genes differs among developmental stages and under various growth conditions.

With the number of characterized laccase enzymes increasing steadily over the last decades, many counterparts were described in the literature with variable properties, which can differ significantly from those previously known for traditional laccases. Laccase-like MCOs (LMCOs) is a term proposed by Reiss et al. [1], to describe a multigenic family of oxidoreductases with especially variable characteristics, mainly related to substrate specificity, thus confining laccases to MCOs that are active against urushiol, an unsaturated alkyl catechol. LMCOs are found in bacteria, fungi, plant, and insects, and are considered to have similar biological functions to laccases, even though their exact role and substrate spectrum is yet to be clarified. Even though laccase-like enzymes show conserved active site residues, where the copper ligands are organized, they may lack other laccase-specific signature sequences. For example, Kumar et al. [11] described a set of conserved consensus sequences L1–L4 in an effort to develop reliable homology tools to identify ‘true’ laccases. LMCOs usually contain these motifs, but with several amino acid substitutions, as shown for one of the few fungal LMCOs described in the literature, *Tt*LMCO1 from *Thermothelomyces thermophila* [12].

Laccase multigene families and LMCOs have been studied from various perspectives, including enzyme characterization, transcriptome and secretome analysis, expression, and biological function studies. Regarding fungal LMCOs, Chen et al. [13] indicated the presence of laccase-like genes in ectomycorrhizal basidiomycetes (*Piloderma byssinum*). LCMOs have also been detected in *Trichoderma reesei* [14] and *Aspergillus niger* genomes [15]. Another phylogenetic analysis of a laccase multigene family was conducted on seven *Auricularia auricula-judae* laccase genes containing the signature sequences L1–L4, indicating their functional differences from other basidiomycete LMCOs [16]. Furthermore, Vasina et al. [17] focused on a laccase multigene family of the basidiomycete *Trametes hirsuta* strain 072, locating five laccase genes which are not only categorized to different clusters within the genus *Trametes*, but their production also appears to depend on the stage of biomass degradation.

In the field of Industrial Biotechnology, the contribution of laccases has been undeniable. Food and beverage processing, bioethanol production, bioremediation, paper production, dye decolorization, textile functionalization, and biosensor construction are only some of the applications where laccases have been proven to be valuable [18–21]. Especially for LMCOs, most of the proposed biotechnological applications focus on dye decolorization [22, 23].

Moreover, the exploitation of laccases is gaining interest in the field of organic synthesis (i.e., as green catalysts). For example, laccases catalyze the synthesis or modification of bioactive compounds, such as antioxidants [24], alkaloids [25], amino acid analogues [26], or bioactive polymers [27–30]. Although the value of high-redox potential laccases in the field of biocatalysis is widely accepted, there are only a few references on the synthetic properties of low-redox LMCOs, such is the case of *Tt*LMCO1 from *T. thermophila* which catalyzed the conversion of 2′,3,4-trihydroxychalcone to 3′,4′-dihydroxy-aurone [12].

The aim of the present study is the isolation and characterization of two novel LCMOs from *Pleurotus citrinopileatus* (Fungi, Basidiomycota), with high potential for biotechnological applications. *Pleurotus* species are known to contain laccase multigene families in their genome [9], while the degradative potential of *P. citrinopileatus* toward lignocellulosic substrates has been previously demonstrated in sugarcane bagasse [31], paper and cardboard [32], and olive oil mill wastewater (OMWW [33]). Two LMCOs were isolated from the culture supernatants of *P. citrinopileatus* grown on OMWW, and they were fully characterized. Proteomic analyses revealed similarities with major secreted laccases in other *Pleurotus* species. Based on their low formal potentials and thus high substrate specificity, this work intends to highlight the synthetic properties of *Pc*Lac1 and *Pc*Lac2, and provide evidence toward their potential use as biocatalysts in the fields of Green Chemistry and Organic Synthesis.

## Results

### Production and purification of laccases

**Fig. 1:**
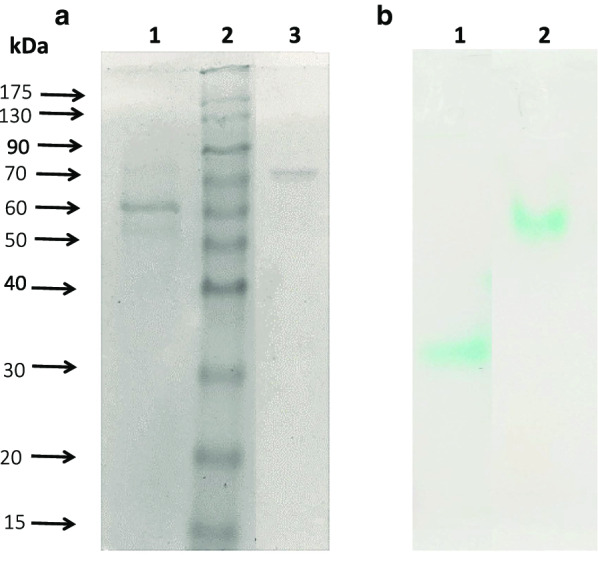
**a** SDS-PAGE analysis of the isolated proteins. Lane 1: *Pc*Lac1, lane 2: protein marker, lane 3: *Pc*Lac2. **b** Zymogram of the isolated proteins, lane 1: *Pc*Lac1, lane 2: *Pc*lac2

For the isolation of putative laccase enzymes from *P. citrinopileatus*, the strain was grown as previously described [33], i.e., in OMWW supplemented with corn steep liquor, for 12 days, until the maximum levels of laccase production were reached, around 1000 U L^−1^. Then, the culture supernatant was collected and concentrated before purification. Purification with Q sepharose column resulted in two separate fractions with laccase activity, which were further purified separately. After an additional purification step with DEAE sepharose, two novel laccases were isolated, named *Pc*Lac1 and *Pc*Lac2. The purification results are shown in Additional file 1: Table S1. Their purity was confirmed with SDS-PAGE analysis and by the respective zymograms (Fig. 1). From the SDS-PAGE, it can be concluded that *Pc*Lac1 is a 60 kDa protein, while *Pc*Lac2 is closer to 75 kDa.

### Proteomic analysis

The identification of in-gel digested protein bands is displayed in Table 1 and Additional file 2. In gel band *Pc*Lac1, 22 MSMS spectra matched with 9 peptides providing 18.6% coverage of the sequence Q2VT18_PLEPU Laccase-2 of *Pleurotus pulmonarius*, which is 96.5% homologous to Q12739 LAC2_PLEOS—Laccase-2 of *Pleurotus ostreatus*.

**Table 1:** Proteomic analysis of PcLac1 and PcLac2

	*Pc*Lac1	*Pc*Lac2	
Description	> tr|Q2VT18|Q2VT18_PLELaccase 2 from *Pleurotus pulmonarius*	> tr|A0A067N2X1|A0A067N2X1_PLEOS Uncharacterized protein from *Pleurotus ostreatus*	> tr|Q2VT19|Q2VT19_PLELaccase 6 from *Pleurotus pulmonarius*
*|Log Prob|*	87.07	69.7	188.3
*Best |Log Prob|"*	17.31	13.4	15.9
*Best score*	985.80	873.2	958.7
*Total Intensity*	4.37E + 08	4.5E + 08	4.1E + 08
*# of PSM spectra*	22	27	65
*# of unique peptides*	9	11	29
*# of mod peptides*	1	3	9
*Coverage %*	18.61	19.5	42.6
*# AA's in protein*	532	591	521
*BLAST homology*	96.5% homologous to Q12739	61% homologous to V2XEX2	99% homologous to A0A067NLM3

Gel band of *Pc*Lac2 displays 92 MSMS spectra matching in total 40 peptides that belong to two different proteins; a laccase from *P. pulmonarius* (Q2VT19_PLEPU laccase 6, 29 peptides with 42.6% sequence coverage) that is 99% homologous to laccase 0A067NLM3_PLEOS from *P. ostreatus* and an uncharacterized protein from *P. ostreatus* (A0A067N2X1_PLEOS, 11 peptides with 19.5% sequence coverage) that is 61% homologous to copper radical oxidase V2XEX2 from *Moniliophthora roreri*.

### Biochemical characterization of laccases

**Fig. 2:**
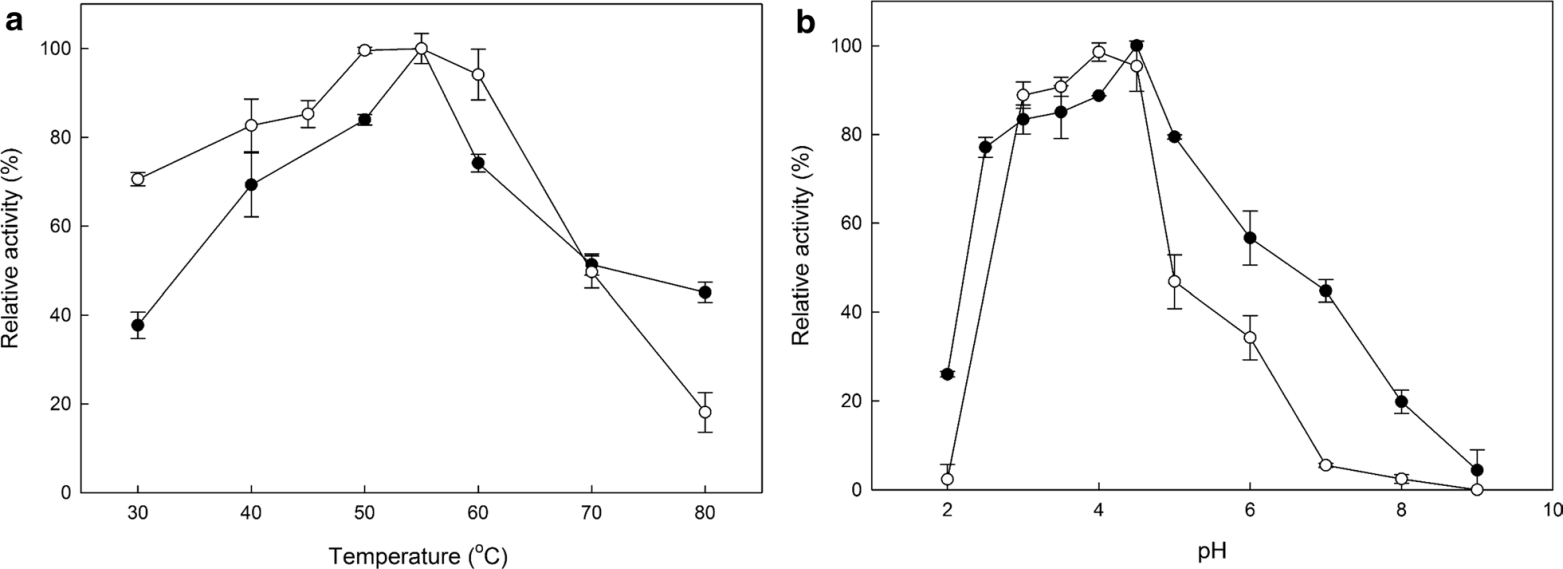
Temperature (**a**) and pH (**b**) optimum for the laccases *Pc*Lac1 (*black circles*) and *Pc*Lac2 (*white circles*)

**Fig. 3:**
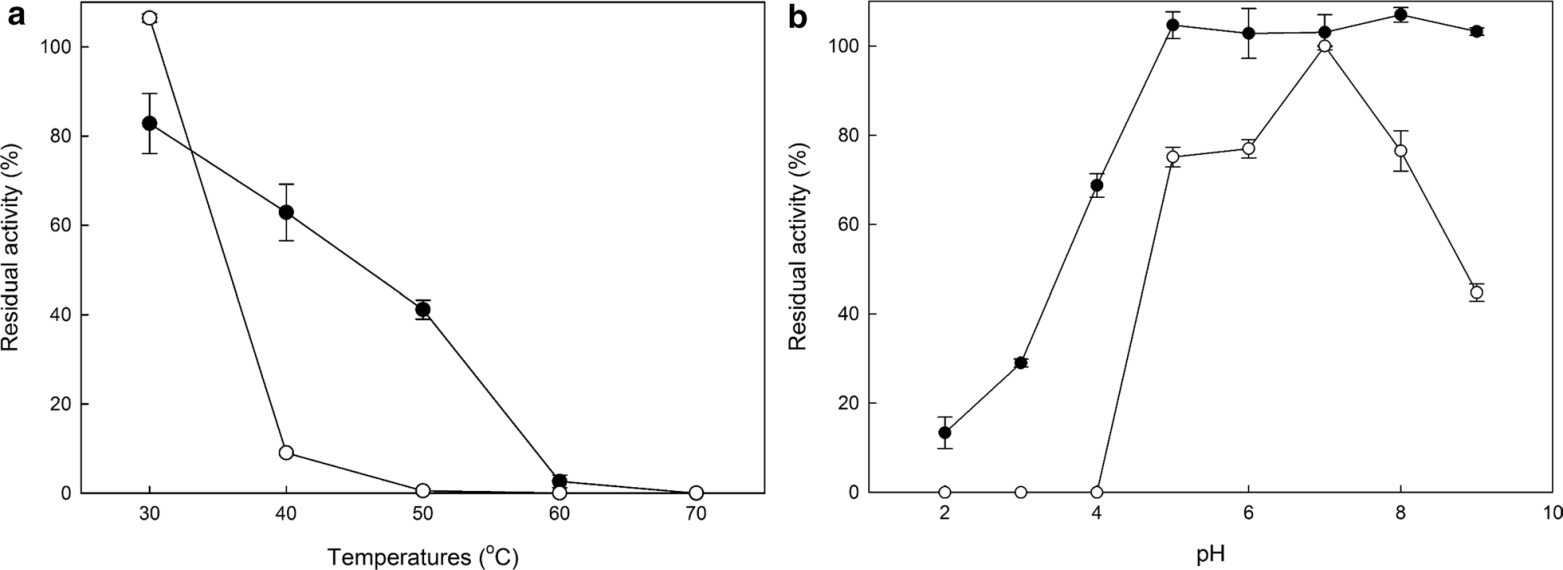
**a** Temperature stability of *Pc*Lac1 (*black circles*) and *Pc*Lac2 (*white circles*) after 4 h incubation **b** pH stability of *Pc*Lac1 (*black circles*) and *Pc*Lac2 (*white circles*) after 24 h incubation

The optimal temperature and pH conditions were investigated for both laccases, and the results are shown in Fig. 2. Both enzymes have a temperature optimum at 55 °C, but while *Pc*Lac2 retains over 90% of its activity in the range 50–60 °C, *Pc*Lac1 retains slightly lower than 80% in this same range. In addition, pH optimum was 4 for *Pc*Lac2 and 4.5 for *Pc*Lac1, while both enzymes retained over 80% of their activity in the range 3–4.5. However, in higher pH, the residual activity dropped abruptly, in pH 5 for *Pc*Lac2 and pH 6 for *Pc*Lac1. Stability of both laccases was also investigated for a range of temperatures and pH values, and the results are shown in Fig. 3. *Pc*Lac1 was more thermostable than *Pc*Lac2, which lost almost all activity after 4 h incubation at temperatures over 40 °C. On the contrary, *Pc*Lac1 retained 60% of its activity after 4 h at 40 °C, and a little over 40% for the same time at 50 °C. Regarding the pH stability, both laccases were stable in neutral pH, but they lose quickly their activity at pH lower than 4. *Pc*Lac1 was also stable at basic pH, but *Pc*Lac2 lost some of its activity at pH 8 or higher.

The substrate oxidation spectrum was investigated for both laccases, against a variety of substrates, as shown in Table 2. From the 24 substrates tested, *Pc*Lac1 and *Pc*Lac2 oxidized unambiguously 8 and 14 substrates, respectively. Both laccases were found to strongly oxidize ABTS, but no activity was detected against ascorbic acid and tyrosine, indicating that the studied laccases do not have ascorbate oxidase or tyrosinase activity. Hydroxybenzenes, such as catechol, hydroquinone, and pyrogallol, were also oxidized by the enzymes, similarly to most known laccases. However, the very low levels of guaiacol oxidation detected for both enzymes are rather unusual for fungal laccases, although both enzymes oxidized 2,6-dimethoxyphenol. Phenol and vanillin were also not oxidized by the studied laccases, indicating a rather low formal potential; however, *Pc*Lac2 activity was demonstrated—to a certain extent—against vanillic acid, veratryl alcohol (3,4-dimethoxybenzyl alcohol), and *p*-cresol. These distinct differences in the substrate oxidation spectrum of the two laccases might indicate a potentially different organization and architecture of the copper cluster site. Moreover, both laccases were found to strongly oxidize most of the hydroxycinnamic acids tested, except *p*-coumaric acid, but only *Pc*Lac2 was able to oxidize hydroxybenzoic acids, such as vanillic and gallic acids. Interestingly, both enzymes strongly oxidized quercetin, which might be an indication of their possible involvement in the production of bioactive compounds and secondary metabolites from *P. citrinopileatus*.

**Table 2:** Substrate oxidation spectrum of *Pc*Lac1 and *Pc*Lac2

	Substrate	*Pc*Lac1	*Pc*Lac2
*Hydroxybenzenes*			
1	Phenol	−	−
2	Catechol	+	+ +
3	Resorcinol	−	−
4	Hydroquinone	+	+
5	Chlorocatechol	−	+
6	Pyrogallol	+	+
*Methoxyphenols*			
7	Guaiacol	±	±
8	2.6-Dimethoxyphenol	+	+
*Aromatic alcohols*			
9	3.4-Dimethoxybenzyl alcohol	−	+
*Phenethyl alcohols*			
10	Tyrosol	−	−
*Aromatic amines*			
11	Aniline	−	−
12	Tyrosine	−	−
*Phenolic aldehydes*			
13	Vanillin	−	−
*Flavonoids*			
14	Quercetin	+ +	+ +
*Hydroxycinnamic acids*			
15	Caffeic acid	+	+
16	Ferulic acid	+	+
17	Sinapic acid	+	+
18	*p*-Coumaric acid	−	−
*Hydroxybenzoic acids*			
19	Vanillic acid	−	+
20	Gallic acid	−	+
*Aromatic azo compounds*			
21	ABTS	+ + +	+ + +
*Other acids*			
22	Ascorbic acid	−	−
*Cresols*			
23	*o*-Cresol	−	−
24	*m*-Cresol	−	−
25	*p*-Cresol	−	+

Activity, in terms of absorbance difference in the recorded UV/Vis spectrum between reaction and blank, is indicated with ( +) when positive (indicating absorbance difference in the spectrum maxima), with (−) when negative and with ( ±) when ambiguous. Multiple ( +) signs indicate differences higher than 1 (+ +) or 2 (+ + +) absorbance units

The substrate specificity was determined for both enzymes against some of the tested substrates, and the results are shown in Table 3. ABTS was the substrate most strongly oxidized by both laccases, but *Pc*Lac2 showed a significantly higher specific activity than *Pc*Lac1. The specificity for the other compounds tested is similar for both enzymes, yet significantly lower. Remarkably, the specific activity of both enzymes against guaiacol was very low, several times lower than in the case of 2,6-DMP, a property rather unusual for laccases from fungal sources.

**Table 3:** Substrate specificity of *Pc*Lac1 and *Pc*Lac2

Substrate	*Pc*Lac1 (Units/mg)	*Pc*Lac2 (Units/mg)
ABTS	1.48 ± 0.09	4.78 ± 0.33
2.6 DMP	0.08 ± 0.00	0.019 ± 0.003
Catechol	0.06 ± 0.01	0.04 ± 0.01
Pyrogallol	0.020 ± 0.001	0.0390 ± 0.0003
Guaiacol	0.003 ± 0.000	0.0006 ± 0.0002
Hydroquinone	0.079 ± 0.003	0.09 ± 0.02

Michaelis–Menten kinetic parameters were calculated for both enzymes on ABTS and 2,6-DMP (Table 4). Both enzymes showed a high affinity for ABTS, similarly to other fungal laccases, but a significantly lower affinity for 2,6-DMP. However, *Pc*Lac1 exhibited a much higher affinity for both substrates compared to *Pc*Lac2. The turnover number was low in the case of 2,6-DMP oxidation, but considerably higher in the case of ABTS oxidation. *Pc*Lac2 showed a very high turnover number for ABTS oxidation in comparison with *Pc*Lac1. The same trend was followed for the catalytic efficiency.

**Table 4:** Michaelis–Menten kinetic parameters calculated for *Pc*Lac1 and *Pc*Lac2

Enzyme	Substrate	*V*_max_ (Units/mg)	*k*_cat_ (min^−1^)	*K*_*M*_ (μΜ)	*K*_cat_/*K*_*M*_ (μM^−1^ min^−1^)
*Pc*Lac1	ABTS	2.38 ± 0.06	95.2 ± 2.3	60 ± 7	1.57 ± 0.17
	2.6-DMP	0.36 ± 0.01	14.2 ± 0.5	672 ± 56	0.021 ± 0.002
*Pc*Lac2	ABTS	4.6 ± 0.1	368.7 ± 8.8	165 ± 15	2.24 ± 0.21
	2.6-DMP	0.041 ± 0.002	3.3 ± 0.2	1070 ± 105	0.0030 ± 0.0003

**Fig. 4:**
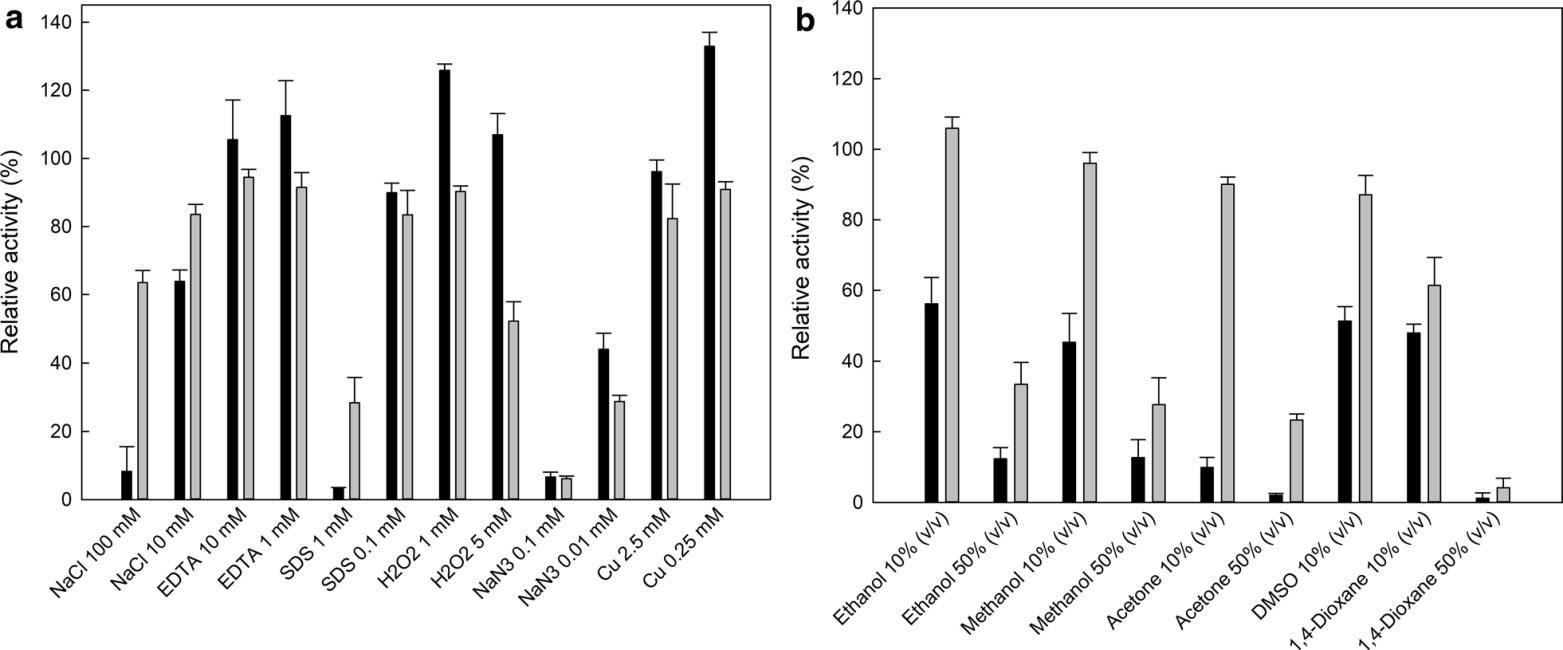
Effect of inhibitors (**a**) and solvents (**b**) on the activity of laccases *Pc*Lac1 (*black bars*) and *Pc*Lac2 (*gray bars*)

The effect of various inhibitors and solvents was also tested on the activity of both *Pc*Lac1 and *Pc*Lac2, and the results are shown in Fig. 4. EDTA, a known inhibitor of laccase activity, did not seem to affect significantly the activity of both enzymes, at least up to the tested concentrations. On the contrary, NaN_3_, also a known laccase inhibitor, highly affected the activity of both enzymes; residual activity was lower than 50% for both enzymes, even with the addition of 0.01 mM NaN_3_. Addition of copper at low concentrations enhanced the activity of *Pc*Lac1, while *Pc*Lac2 was not affected. NaCl strongly inhibited the activity of *Pc*Lac1, but not of *Pc*Lac2. Regarding the effect of solvents, *Pc*Lac1 was strongly affected in most cases, since the residual activity was lower than 60% in all conditions tested. On the other hand, *Pc*Lac2 was found to be more resistant to the presence of solvents. The residual activity observed was higher than 80% with the addition of 10% of either ethanol, methanol, acetone, and DMSO, and over 60% of initial activity was observed with the addition of 10% 1,4-dioxane. The satisfactory stability of *Pc*Lac2 in the presence of solvents might be an attractive property for biocatalysis applications, especially in biotransformations of phenolic compounds, where the low water solubility of both substrates and products requires the use of organic solvents.

### Determination of redox potential

FTacV was performed for *Pc*Lac1 and *Pc*Lac2 at different temperatures *T* and frequencies *f*, while keeping the scan rate *v* and the amplitude *A*_*0*_ steady at 50 mV/s and 180 mV, respectively. The temperatures examined were 30, 33, 39, and 43 °C, and the frequencies corresponded to values of 6, 9, 12, and 16 Hz. The 3rd harmonic for the different frequencies at an indicative temperature of 43 °C is presented in Additional file 1: Figure S1. In the third harmonic, the signal is already clear enough with no strong effect of capacitance currents, and thus, a value for the formal potential *E*^0′^ of the active site of the enzymes can be determined without having to resort to higher order of harmonics. The potential is calculated from the dominant peak of peak triplets that appear around 250 mV for *Pc*Lac1 and 180 mV vs Ag|AgCl, KCl sat. for *Pc*Lac2 (Table 5). An average value of the formal potential from the different frequencies was calculated at each temperature together with the respective standard deviation. From the pattern of the 3rd harmonics, it can be assumed that the reduction and oxidation of these enzymes fall into the quasi reversible case, where the value of the dominant peak corresponds to the value of the formal potential [34]. The smaller irreversible peak that appeared at more negative potentials in both cases is not attributed to the enzyme but to other impurities existing in the samples due to incomplete purification and, thus, should not be considered for the present study.

**Table 5:** Calculated and corrected values of the apparent formal potential of *Pc*Lac1 and *Pc*Lac2 at different temperatures

*T* (°C)	*E*^0′^ vs Ag|AgCl KCl sat. (mV)	*E*^0′^_corrected_ vs Ag|AgCl KCl sat. (mV) corrected	*E*^0′^_corrected_ vs NHE (mV)	Standard deviation
*Pc*Lac1				
30	261	256	453	1.2
33	262	257	454	0.7
39	260	255	452	1.0
43	270	265	462	7.8
*Pc*Lac2				
30	182	177	374	3.9
33	187	182	379	2.8
39	188	183	380	1.8
43	182	177	374	10.1

Regarding the variance of the formal potential with the temperature, it can be concluded that in the temperature range examined, no considerable effect was observed.

### Biocatalytic synthesis of phenolic acid derivatives

*Pc*Lac1 and *Pc*Lac2 were applied to oxidation reactions of ferulic acid and sinapic acid, with the aim to investigate their potential at oxidative oligomerization of phenolic acids. Samples were drawn at 3 h and 8 h intervals, and analyzed with UHPLC–HRMS/MS. After 24 h reaction, the formation of precipitate was observed, indicating the polymerization of the substrates to insoluble compounds. The reaction mixtures were centrifuged and the supernatant was also analyzed by UHPLC–HRMS/MS.

The results for the bioconversion of ferulic acid are shown in Table 6. The double decarboxylated dimer **1** was found in all samples of *Pc*Lac2 as a major constituent, and was detected in *Pc*Lac1 samples in traces only. The decarboxylated dimer could be detected in all samples, 3 isomers at Rt 7.07, 7.29, and 7.99 min (**2, 4, 5**). The dimer **3** was found in all samples. In *Pc*Lac1 samples, the dimer was major, while in *Pc*Lac2 samples, the decarboxylated dimers were the main constituents. In all samples, trimers were the less intense peaks.

**Table 6:** LC–MS identification of the main ions detected during oxidation of ferulic acid by *Pc*Lac1 and *Pc*Lac2

Compound	Rt (min)	m/z [M-H]^−^	MF	MS/MS	Identification	*Pc*Lac1 samples	*Pc*Lac2 samples
*Dimers*							
**1**	7.07	297.111	C_18_H_17_O_4_	146 (C_9_H_6_O_2_) 109 (C_6_H_5_O_2_)	Double-decarboxylated diferulic acid	Minor	Major
**2**	7.07	341	C_19_H_17_O_6_	281 (C_17_H_13_O_4_), 267 (C_16_H_11_O_4_), 209 (C_14_H_9_O_2_), 159 (C_10_H_7_O_2_), 146 (C_9_H_6_O_2_)	Decarboxylated diferulic acid	Major	Major
**3**	7.28	385	C_20_H_17_O_8_	267 (C_16_H_11_O_4_), 239 (C_15_H_11_O_3_)	Diferulic acid	Major	Major
**4**	7.29	341	C_19_H_17_O_6_	281 (C_17_H_13_O_4_), 267 (C_16_H_11_O_4_), 209 (C_14_H_9_O_2_), 159 (C_10_H_7_O_2_), 146 (C_9_H_6_O_2_)	Decarboxylated diferulic acid	Major	Major
**5**	7.99	341	C_19_H_17_O_6_	281 (C_17_H_13_O_4_), 267 (C_16_H_11_O_4_), 209 (C_14_H_9_O_2_), 159 (C_10_H_7_O_2_), 146 (C_9_H_6_O_2_)	Decarboxylated diferulic acid	Major	Major
*Trimers*							
**6**	7.40	577	C_30_H_25_O_12_	193	Triferulic acid	Not detected	Major
**7**	7.62	533	C_29_H_26_O_10_	193	Triferulic acid, decarboxylated	Not detected	Major
**8**	7.63	577	C_30_H_25_O_12_	193	Triferulic acid	Not detected	Major
**9**	7.65	533	C_29_H_26_O_10_	193 (C_10_H_9_O_4_),	Triferulic acid, decarboxylated	Minor	Major
**10**	7.67	533	C_29_H_26_O_10_	193	Triferulic acid, decarboxylated	Not detected	Major
**11**	8.02	533	C_29_H_26_O_10_	193	Triferulic acid, decarboxylated	Minor	Major
**12**	8.21	533	C_29_H_26_O_10_	193	Triferulic acid, decarboxylated	Minor	Major
**13**	8.35	533	C_29_H_26_O_10_	193	Triferulic acid, decarboxylated	Minor	Major
**14**	8.35	565	C_30_H_29_O_11_	193 (C_10_H_9_O_4_), 178 (C_9_H_6_O_4_), 149 (C_9_H_9_O_2_), 134 (C_8_H_6_O_2_)	Unknown triferulic acid	Major	Major
**15**	8.63	533	C_29_H_26_O_10_	193	Triferulic acid, decarboxylated	Not detected	Minor
Others							
**16**	7.5	551	C_29_H_28_O_11_	193 (C_10_H_9_O_4_), 178 (C_9_H_6_O_4_), 149 (C_9_H_9_O_2_), 134 (C_8_H_6_O_2_)	Triferulic acid, decarboxylated-H_2_O*	Detected	Detected

*The MS/MS shows that it is a triferulic acid derivative but it is not obvious how. A carboxyl group and a H_2_O molecule are missing

The mono decarboxylated triferulic acid comes out to 4 isomers **9**, **11**, **12,** and **13**, corresponding to Rt 7.65, 8.02, 8.21, and 8.35 min, respectively. These trimers are most probably position isomers, as they share similar fragmentation patterns. Their structure was confirmed by the MS/MS spectra with a fraction at 193 m/z corresponding to the ferulic acid ion. They were recorded in all samples, but they were less intense in *Pc*Lac1 samples. In addition, three other isomers (**7**, **10**, and **15)** were detected in *Pc*Lac2 samples at Rt 7.62, 7.67, and 8.63 min, respectively. Two isomers of triferulic acid with m/z 577, C_30_H_25_O_12_ (**6** and **8**) were detected in *Pc*Lac2 samples, with Rts 7.40 and 7.63, respectively.

The results for the bioconversion of sinapic acid are shown in Table 7. The decarboxylated dimer **19** was found in *Pc*Lac2 but not in *Pc*Lac1 samples. The dimer **18** was found in *Pc*Lac1 samples corresponding to 3 and 8 h of reaction, and not in *Pc*Lac2 reaction. Trimer **22** at m/z 667 Rt 7.37 were found in *Pc*Lac2 samples in very small quantities. The decarboxylated trimers **23** and **24** at m/z 623 at Rts 7.10 and 7.56, respectively, were found in *Pc*Lac2 reactions only and not in the corresponding *Pc*Lac1. A di-decarboxylated trimer (**25**) at m/z 579 at 8.16 min was found only in *Pc*Lac2 reactions and was absent from *Pc*Lac1 ones. Furthermore, a tetramer of the sinapic acid (**26**) was detected in *Pc*Lac2 samples.

**Table 7:** LC–MS identification of the main ions detected during oxidation of sinapic acid by *Pc*Lac1 and *Pc*Lac2

Compound	Rt (min)	m/z [M-H]^−^	MF	MS/MS	Identification	*Pc*Lac1 samples	*Pc*Lac2 samples
*Dimers*							
**17**	6.19	445	C_22_H_21_O_10_	189 (C_11_H_9_O_3_), 174 (C_10_H_6_O_3_), 161 (C_9_H_5_O_3_), 145 (C_9_H_5_O_2_), 121 (C_7_H_5_O_2_)	Disinapic acid	Minor	Major
**18**	6.69	445	C_22_H_21_O_10_	189 (C_11_H_9_O_3_), 174 (C_10_H_6_O_3_), 161 (C_9_H_5_O_3_), 145 (C_9_H_5_O_2_), 121 (C_7_H_5_O_2_)	Disinapic acid	Major	Minor
**19**	6.97	401.1220	C_21_H_21_O_8_	189 (C_11_H_9_O_3_), 174 (C_10_H_6_O_3_), 161 (C_9_H_5_O_3_), 145 (C_9_H_5_O_2_), 133 (C_8_H_5_O_2_), 121 (C_7_H_5_O_2_)	Decarboxylated disinapic acid	Not detected	Detected
**20**	7.21	401.1220	C_21_H_21_O_8_	189 (C_11_H_9_O_3_), 174 (C_10_H_6_O_3_), 161 (C_9_H_5_O_3_), 145 (C_9_H_5_O_2_), 133 (C_8_H_5_O_2_), 121 (C_7_H_5_O_2_)	Decarboxylated disinapic acid	Not detected	Detected
**21**	6.26	357.060	C_18_H_13_O_8_	271 (C_14_H_7_O_6_), 227 (C_13_H_7_O_4_), 199 (C_12_H_7_O_2_), 167 (C_12_H_7_O)	Bis-Decarboxylated disinapic acid	Detected	Detected
*Trimers*							
**22**	7.38	667.1643	C_33_H_31_O_15_	-	Trisinapic acid	Not detected	Minor
**23**	7.09	623.1746	C_32_H_31_O_13_	265 (C_16_H_9_O_4_), 209 (C_14_H_9_O_2_), 121 (C_7_H_5_O_2_)	Trisinapic acid, decarboxylated	Not detected	Detected
**24**	7.55	623.1746	C_32_H_31_O_13_	265 (C_16_H_9_O_4_), 209 (C_14_H_9_O_2_), 121 (C_7_H_5_O_2_)	Trisinapic acid, decarboxylated	Not detected	Detected
**25**	8.16	579.1846	C_31_H_31_O_11_	339 (C_19_H_15_O_6_)	Trisinapic acid, bis-decarboxylated	Not detected	Detected
*Tetramers*							
**26**	6.98	891.2319	C_44_H_43_O_20_	341 (C_19_H_17_O_6_), 189 (C_11_H_9_O_3_), 174 (C_10_H_6_O_3_), 161 (C_9_H_5_O_3_), 145 (C_9_H_5_O_2_), 121 (C_7_H_5_O_2_)	Tetrasinapic acid	Not detected	Detected
Others							
**27**	7.99	503.1172	C_24_H_23_O_12_	147 (C_8_H_3_O_3_) 119 (C_7_H_3_O_2_)	Unknown compound	Detected	Not detected

In the bioconversion reactions for both ferulic and sinapic acid, the samples corresponding to 24 h reaction with *Pc*Lac1 seemed to be degraded, since only a few of the detected compounds were found in this case. This might be attributed to further polymerization of the oligomers produced in the first hours of the reaction, and their subsequent precipitation from the reaction mixture.

## Discussion

In most fungal genomes, many copies of laccase-encoding genes are present, forming laccase multigene families. In the genome of *P. ostreatus,* 12 laccase genes have been detected, but only 6 of them have been isolated and characterized [8, 9]. Therefore, there is still a significant knowledge gap on the minor laccase enzymes produced by *Pleurotus* species, which nonetheless contribute in a major way to the decomposition of complex plant materials. A study regarding *P. ostreatus* genome identified 3 laccase gene groups [10], while the isolated laccase isozymes showed high variability in terms of ABTS oxidation and malachite green decolorization. The same study revealed, apart from the existence of major laccase isozymes with potent dye decolorization activity, the presence of minor laccase isozymes. The genes *lacc2* and *lacc11*, and the corresponding enzymes, were considered ‘defective’ laccases by the authors, due to the absence of the characteristic HXHG and copper-binding HCH motifs, respectively. Lacc2 (POXA3) and Lacc11 did not decolorize malachite green, and their expression in the tested conditions was low, although Castanera et al. [9] reported enhanced expression of Lacc2 in wheat straw-based media. Consequently, their biochemical properties and physiological role were not examined, although their presence in *P. ostreatus* transcriptome is established. The laccase system of *P. citrinopileatus* seems to be similar to *P. ostreatus*, since the two laccases isolated from the supernatant of *P. citrinopileatus* cultures accounted for 50% of total laccase activity. Furthermore, *Pc*Lac1 was found to be similar to LACC2 from *P. ostreatus*, and *Pc*Lac2 was also found to be similar to another *P. ostreatus* laccase. The relatively low E^0′^ and narrow substrate range of *Pc*Lac1 together with the previously studied biochemical properties of LACC2 of *P. ostreatus* indicate that the laccase-based ligninolytic system of the two *Pleurotus* species has many similarities.

In our previous work regarding the degradation of phenolic compounds from OMWW by *P. citrinopileatus*, we obtained laccase zymograms on both SDS-PAGE and IEF gels with large smear bands, possibly corresponding to multiple laccase enzymes [33]. In the present study, we aimed at the isolation and characterization of laccase isozymes during the growth of the fungus in OMWW. The fractionation of the culture supernatant at the selected conditions resulted in the isolation of two separate peaks with laccase activity. The corresponding enzymes were named *Pc*Lac1 and *Pc*Lac2. Both enzymes correspond to slightly over 50% of the total laccase activity of the crude supernatant, indicating the existence of other laccases, which were not isolated by the selected chromatographic conditions (Additional file 1: Table S1).

The molecular mass of both *Pc*Lac1 and *Pc*Lac2 lies within the range of most fungal laccases [3]. They both have acidic pH optima, but *Pc*Lac1 was found more thermostable and pH-stable than *Pc*Lac2. In addition, the substrate range of the studied laccases shows some interesting features. Both laccases were able to oxidize *ortho*- and *para*-substituted phenols (catechol and hydroquinone), but not *meta-* substituted phenols (resorcinol), similarly to what was reported for many microbial laccases [1]. The existence of *ortho*- or *para*- substituents facilitates the electron abstraction from the phenoxy-OH, leading to the formation of the respective radical. As shown previously [35], the apparent *K*_*m*_ of laccases is the result of both the enzyme’s affinity for the substrate and the substrate electric properties, such as the redox potential and the ionization energy [1]. An interesting observation regarding the substrate preference of the studied laccases is that they both show higher specific activity for 2,6-DMP than the mono-methoxy- substituted guaiacol. This is unusual for fungal laccases, since guaiacol is considered one of the traditional substrates. In this respect, *Pc*Lac1 and *Pc*Lac2 are more similar to bacterial laccases, which lack the ability to oxidize guaiacol [1]. Both laccases can oxidize hydroxycinnamic acids, except *p*-coumaric acid, but only *Pc*Lac2 can oxidize hydroxybenzoic acids, such as vanillic and gallic acid. This is an interesting observation, since most known fungal laccases have the ability to oxidize gallic acid. Together with the fact that *Pc*Lac2 is able to oxidize vanillic acid, but not vanillin, we may assume the presence of a residue interacting with the carboxyl group, corresponding to Asn264 of *T. versicolor* laccase [35], and thus facilitating the electron abstraction from substrates bearing the –COOH group. The same authors attributed the oxidation of substrates bearing ortho- substituted methoxy or hydroxyl groups, to the hydrogen bonds formed with His458 and Asp206 in *T. versicolor* laccase, which stabilize the substrate and facilitate electron abstraction. The kinetic constants calculated for *Pc*Lac1 and *Pc*Lac2 are also in the range reported for other fungal laccases [3], and specifically for *Pleurotus* species. Regarding ABTS oxidation, *Pc*Lac2 showed a similar *K*_*m*_ value to Lacc6 from *P. ostreatus* [10], while the *K*_*m*_ calculated for *Pc*Lac1 on ABTS was similar to two laccases isolated from *P. ostreatus* by Tinoco et al. [36]. These biochemical properties are in accordance with the proteomics data, which illustrate the similarity of *P. citrinopileatus* laccase system with the corresponding enzymatic system of *P. ostreatus*.

Regarding the effect of various inhibitors on the activity of *Pc*Lac1 and *Pc*lac2, the results were similar to previously described fungal laccases. The addition of low copper concentrations enhanced *Pc*Lac1 activity, but no effect was observed on the activity of *Pc*Lac2. While many studies reported no effect on laccase activity in the presence of low copper concentrations [20], others reported a certain degree of activity enhancement [37]. This is attributed to the variable architecture of laccase molecules, allowing a certain degree of mobility to the copper ions for some laccases, as in the case of *Coprinus cinereus* laccase, which is able to lose reversibly the Cu ions in the T2 copper center [38]. Nonetheless, increasing Cu concentration above a certain point usually inhibits laccase activity [20], similarly to *Pc*Lac1 and *Pc*Lac2. On the other hand, NaN_3_ strongly inhibited the activity of *Pc*Lac1 and *Pc*Lac2, similarly to most known multicopper oxidases [12, 20]. NaN_3_ binds to the copper atoms of laccases, disrupting the electron transfer.

Regarding the effect of organic solvents, *Pc*Lac2 was found to be significantly more stable than *Pc*Lac1. The stability of *Pc*Lac2 is high in the presence of 10% of most studied solvents, similar to LACC6 from *P. ostreatus* [20]. On the other hand, *Pc*Lac1 was found to be severely inhibited by most organic solvents tested, similarly to most fungal laccases. Solvent stability is an important property for oxidases in biocatalysis applications, since the solubility of most phenolic compounds used as substrates for laccases is limited in aqueous-based media. This is reflected in the efforts made—through genetic engineering—to increase their stability in the presence of organic solvents [39–41].

The formal potential of *Pc*Lac1 and *Pc*Lac2 was calculated with FtacV. *Pc*Lac1 was found to have a higher E^0′^ than *Pc*Lac2, a result that is in agreement with the substrate spectrum. Overall, the electrochemical and biochemical data indicated a rather higher oxidative activity for *Pc*Lac1. Temperature variations between 30 and 43 °C did not seem to affect significantly the E^0′^ of both enzymes. The low-E^0′^ values measured for both laccases are rather unusual for oxidative enzymes from white-rot fungi, since most low-E^0′^ laccases reported in the literature are from bacterial or plant sources [1]. Indeed, significant research effort has been dedicated in the quest for high-E^0′^ oxidative enzymes, due to their outstanding performance in biodegradation and detoxification applications, targeting recalcitrant pollutants. However, their use as biocatalysts for ‘green’ synthetic applications has always been hindered by the formation of multiple products, which creates the need for further costly purification steps. Moreover, the use of laccases as tools for biosensor development suffers from the same issue: their wide substrate specificity results in biosensors with low selectivity against target compounds. The discovery of novel laccases with low E^0′^ could offer a viable alternative to these issues, by providing an array of enzyme options with variable properties, tailored for specific applications.

Regarding the biocatalytic potential of *Pc*Lac1 and *Pc*Lac2 enzymes, the bioconversion of ferulic and sinapic acid was studied in 24 h experiments. It should be noted that the stability profiles of both *Pc*Lac1 and *Pc*Lac2 against 10% (v/v) DMSO enabled the addition of this solvent to the bioconversion reactions, thus allowing the introduction of higher concentrations of the starting monomers. This property can be exploited in ‘green’ synthesis applications, involving substrates with low water solubility. Both enzymes were shown to polymerize the substrates, since the formation of precipitates was observed in all cases at the end of the reaction. The water-soluble reaction products were analyzed with UHPLC–HRMS/MS. The product profiles of the two enzymes presented clear differences, reflecting their difference in redox, biochemical and kinetic properties. The most obvious difference between the two enzymes is that the substrates seem to be polymerized from *Pc*Lac1, and therefore, they precipitate out of the reaction in prolonged reaction times.

The study of Slagman et al. [42], regarding the oligomerization of 4-hydroxybenzoic acid by *T. versicolor* laccase, concluded that not all dimers formed by the action of the enzyme are the same. Some of them can be oxidized by the laccase easier than the original monomer, but other dimers might be less reactive, and thus, they are recovered in higher yields. These conclusions may be applicable in the present study, since the radicals generated by laccase oxidation usually react further in a non-enzymatically controlled fashion. Under this view, *Pc*Lac1 and *Pc*Lac2 should theoretically show similar product profiles. The greater abundance of phenolic acid oligomers shown by *Pc*Lac2 is, therefore, the result of a lower redox potential, since these oligomers are rapidly polymerized by *Pc*Lac1, and precipitate out of the reaction. These results might be of great significance for applications involving environmentally friendly synthesis routes for novel compounds with significant biotechnological potential.

**Fig. 5:**
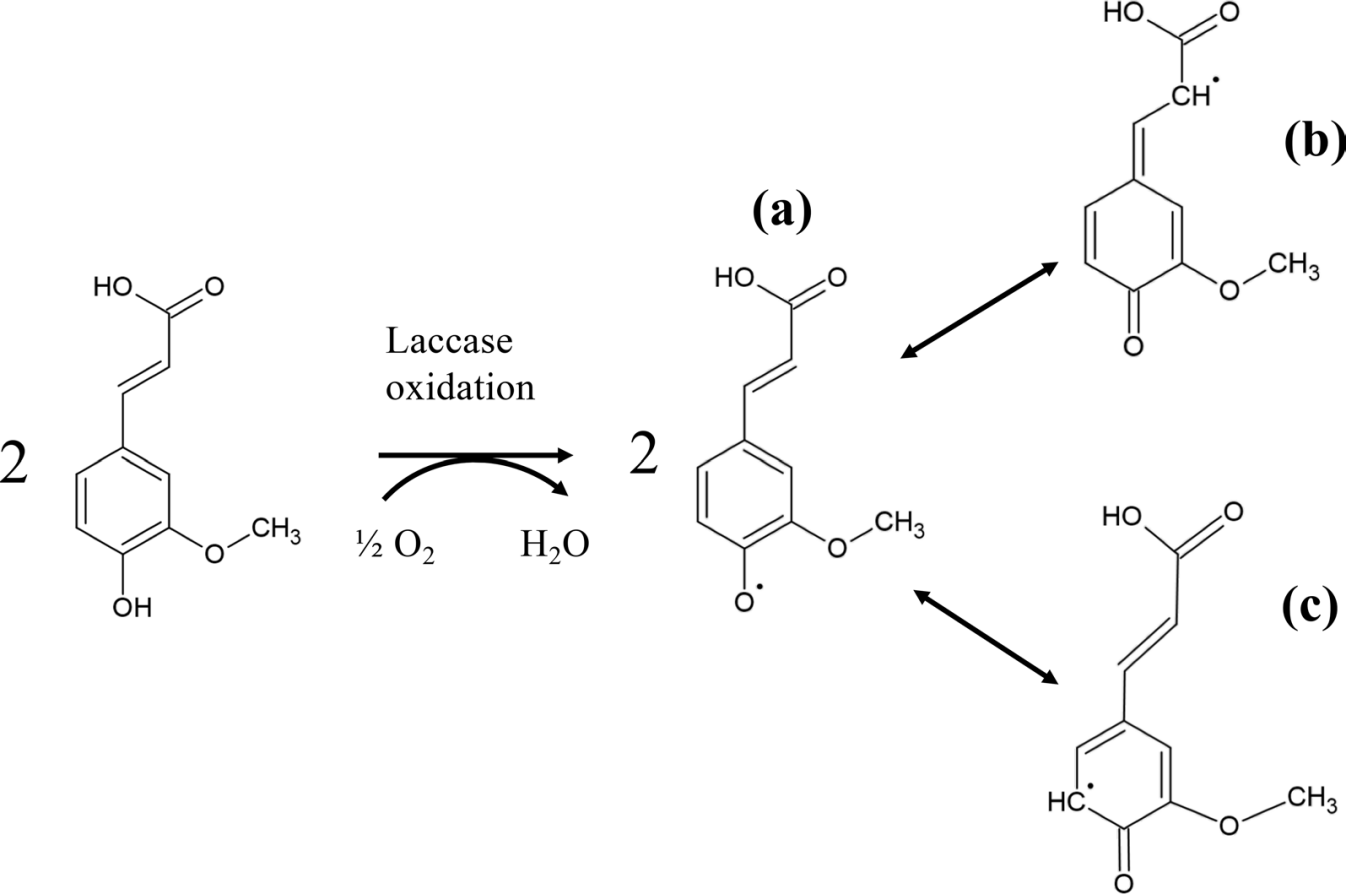
Laccase-mediated oxidation of hydroxycinnamic acids. The O• radical (**a**) is formed by the action of laccase, resulting in the intermediate radicals β (**b**) and 5 (**c**), while only the radicals that contribute to bond-forming events are shown. The R could be either a hydrogen for ferulic acid, or a methoxy group for sinapic acid. The coupling of these intermediates results in the formation of β-β, β-5 β-Ο-4, 5–5, 5-O-4, and O–O dimers. The O–O coupled dimer is unstable, and thus, it is not found among the products, while for sinapic acid oxidation, C5 is occupied by a methoxy group, and therefore does not participate in bond forming [47, 48]

Significant research efforts have been dedicated to the biological activity of hydroxycinnamic acids [43–45], fully documenting their antioxidant, anticancer, and radical-scavenging activity. Enzymatic synthesis, if implemented in a controlled fashion, could provide novel bioactive molecules with superior properties. Ferulic acid dimers are already tested as possible therapeutic agents [46]. As regards oxidative biotransformations, most characterized laccases are relatively high-redox enzymes, capable of oxidizing a great variety of phenolic compounds. This property is favorable for many applications, such as biodegradation of xenobiotic compounds, but it can pose many problems in biocatalytic applications, due to the formation of highly oxidized, variable products. It is generally acknowledged that the redox potential plays a major role in the substrate selectivity of laccases [35], although substrate affinity is also a significant property. Therefore, low-E^0′^ oxidative enzymes could offer an interesting alternative in the biocatalytic synthesis of novel compounds with biological activity. In the present study, the production of novel derivatives of hydroxycinnamic acids was shown, including trimer and tetramer compounds. The general laccase-mediated oxidation scheme of hydroxycinnamic acids is shown in Fig. 5, while the possible oligomerization products previously described in the literature are shown in Additional file 1: Figures S2–S5. Dimerization of hydroxycinnamate acids can lead to the formation of 5′-5′ dimers (Additional file 1: Figure S2, compounds **6** and **10**, Additional file 1: Figure S3, compound 6), 5-*O*-4′ dimers (Additional file 1: Figure S2, compounds **2**, **7** and **9**, Additional file 1: Figure S3, compound **12**), β-5 dimers (from 8, 5′ dimerization, Additional file 1: Figure S2, compound **3**, Additional file 1: Figure S3, compound **10**), 8-*O*-4′ dimers (Additional file 1: Figure S3, compound **9**), or 8,8′ diacids, which can be transformed to either monolactone or dilactone β–β dimers (Additional file 1: Figure S3, compounds **5**, **7** and **8**) [47, 48]. Hydroxycinnamate oligomers have been chemically synthesized and/or isolated from plants, such as the ferulic acid trimer with mass of 578, isolated from maize bran (Additional file 1: Figure S4, compound **10**; [49]).

Studies reporting the enzymatic synthesis of hydroxycinnamate oligomers are sparse. Ward et al. [50] reported the lignin peroxidase-mediated synthesis of three major ferulic acid dimers (Additional file 1: Figure S3, compounds **5**, **8**, and **10**) and two trimers (Additional file 1: Figure S4, compounds **1** and **3)**, one of which showed the same *m/z* (552) (Additional file 1: Figure S4, compound **3**) with one of the trimers obtained in this study (Table 6, compound **16**). The synthesis of one sinapic acid tetramer was previously reported [51] by a plant peroxidase, together with a variety of dimers. The characterized tetramer had *m/z* 831.25 and molecular formula C_43_H_43_O_17_, which is of different molecular mass than the tetramer reported in this study (Table 7, compound **26**). Adelakun et al., [24], also reported the laccase-mediated dimerization of ferulic acid in products with MW of 386 (Additional file 1: Figure S3, compounds **8** and **10**), similar to product **3** reported here (Table 6).

## Conclusions

In the present work, the discovery, purification, and characterization of two new low-redox potential laccases is described, from the white-rot fungus *Pleurotus citrinopileatus*. Peptide analysis of the purified proteins revealed similarities with the laccase system of *P. ostreatus*, but this is the first report of their characterization. The satisfactory stability of both enzymes against different organic solvents allowed the use of DMSO in the biotransformation reactions of ferulic and sinapic acids, resulting in improved solubility of the starting monomers. Both laccases are able to oligomerize ferulic and sinapic acid to different degrees, revealing the importance of the low-redox potential laccases in biocatalytic applications where high selectivity is required. Moreover, to the best of our knowledge, this is the first report regarding the enzymatic production of sinapic acid trimers and tetramers. Further studies are necessary to determine their exact structure and bioactivity, but the importance of low-redox enzymes in synthetic chemistry is hereby demonstrated.

## Materials and methods

### Chemicals and enzymes

2,2′-Azino-bis-(3-ethylbenzthioazoline-6-sulfonic acid) diammonium salt (ABTS) was from Sigma-Aldrich (St. Louis, MO, USA). Other substrates and chemicals were from Applichem (Darmstadt, Germany) or Sigma-Aldrich, and were of the highest purity available.

### Microorganism and growth conditions

The strain *P. citrinopileatus* LGAM 28684 was previously evaluated in respect to the production of ligninolytic enzymes [52]; it is routinely maintained in Potato Dextrose Agar (PDA; Applichem, Germany), and is stored in the fungal culture collection of the Laboratory of General and Agricultural Microbiology (Agricultural University of Athens). *P. citrinopileatus* was cultivated in an olive mill wastewater-based liquid medium, as described by Zerva et al. [33]. Olive mill wastewater (OMWW) was obtained from an olive oil mill with a three-phase decanter in Kalamata (Peloponnese, S.W. Greece) and was maintained at -20 °C; OMWW composition and pretreatment process prior to fungal growth were as previously described [33]. *P. citrinopileatus* was grown in 50% (v/v) OMWW supplemented with 3 g L^−1^ corn steep liquor as nitrogen source, for 25 days, and then, the supernatant was collected and used for the isolation of laccase enzymes.

### Enzyme purification

The culture supernatant was collected by centrifugation, filtrated, and concentrated using an Amicon ultrafiltration device Stirred Cell 8400 (membrane cutoff 10 kDa, Merck Millipore). The concentrate was dialyzed against 20 mM piperazine buffer pH 5.7 at 4 °C overnight, and applied to an equilibrated Q sepharose column with a flow of 4 mL min^−1^. A linear gradient from 0 to 500 mM NaCl was applied with a Biorad Econo gradient pump. Eluted fractions were analyzed for laccase activity, and selected positive fractions were pooled together, and dialyzed again. A second purification step followed, applying the pooled and dialyzed fractions to a properly equilibrated DEAE-Sepharose column. Elution was performed with a linear gradient from 0 to 500 mM NaCl as previously described. Fractions with laccase activity were pooled, concentrated, and dialyzed against 20 mM Bis–Tris buffer pH 7. Sodium dodecyl sulfate-polyacrylamide gel electrophoresis (SDS-PAGE) was performed according to Laemmli [53], with 12.5% polyacrylamide gels.

### Proteomic analysis

Protein bands were excised from a Coomassie Brilliant Blue (CBB) stained gel. Gel slices were cut into small 1 mm^3^ cubes and transferred to 1.5 mL Eppendorf tubes. Proteins in the gel cubes were digested with a standard “in-gel digestion” protocol, as follows; gel pieces were washed twice with 0.1 M NH_4_HCO_3_:acetonitrile (1:1) and subsequently incubated in 50 μL of 10 mM DTT in 0.1 M NH_4_HCO_3_ (15 min at 56 °C). Afterward, gel pieces were washed with 0.1 M NH_4_HCO_3_:acetonitrile (1:1) and incubated with 50 μL of 55 mM iodoacetamide in 0.1 M NH_4_HCO_3_ (30 min at room temperature in the dark). Subsequently, the buffer was removed, and gel pieces were washed with 0.1 M NH_4_HCO_3_: acetonitrile (1:1) and finally with 100% acetonitrile for 5 min. Gel pieces were dried under vacuum for 20 min and rehydrated in digestion buffer with sequencing grade modified porcine trypsin (Promega; 12.5 ng/µL trypsin in 0.1 M NH_4_HCO_3_) at 37 °C overnight. Peptides were extracted by water bath sonication incubating with 50 μL water, adding 50 μL 50% acetonitrile 0.1% formic acid to the gel and incubating 15 min, recover supernatant and pool with first extract. Pooled peptide extracts were dried by SpeedVac and re-dissolved in 20 μL 0.1% formic acid.

From the re-dissolved peptide extract, 5 μL were injected on a nanoLC-MS system (EASY-LCII connected to Qexactive^PLUS^, ThermoScientific, USA). Peptides were trapped on a 2 cm × 0.1 mm C18 trap column, and separated on an 8 cm × 0.75 mm C18 analytical column (PepSep, Denmark) using a flow rate of 300 nL/min. Sample loading and trapping were performed in buffer A (0.1% formic acid in water), and elution was performed with a 20 min gradient going from 2 to 30% buffer B (0.1% formic acid in acetonitrile), followed by column regeneration at 80% buffer B and re-equilibration at 2% buffer B. The nanoLC eluate was directly sprayed into the source of Qexactive by Flex-ion nanospray, using PepSep nanospray needle at 2.3 kV ESI potential. MS acquisition was performed using a DDA method with alternating MS1 scan at resolution 70,000 profile mode, AGC target 3e6, maxIT 50 ms, scan range 500–1400 m/z, and subsequently 8 MS2 scans centroid mode, resolution 17500 AGC target 5e4, maxIT100 ms, with isolation window 1.6 m/z at NCE = 28 on with preferred peptide match ions of charges 2, 3, or 4 and a dynamic exclusion window of 30 s. Protein identification was performed using the database matching algorithm of Byonic (ProteinMetrics, Cupertino, USA) using either the Uniprot entries of *Pleurotus* (taxon 5320, downloaded 8nov 2019).

### Enzymatic assays

Laccase activity was routinely assayed with ABTS as the substrate (2 mM, ε_420_ = 36,000 M^−1^ cm^−1^), in 50 mM phosphate—citrate buffer pH 4 at 35 °C. The reaction was monitored for 10 min in a Spectra Max 250 microplate reader (Molecular Devices, CA, USA). The ability of both enzymes to oxidize different substrates was explored in 24 h reactions. The UV/Vis spectra (250–750 nm) of the reactions and their respective controls with heat-inactivated enzyme were recorded, to determine differences in absorbance maxima. Substrate specificity assays were performed by measuring laccase activity against a variety of substrates (2 mM), including catechol (*ε*_450_ = 928 M^−1^ cm^−1^), 2,6-dimethoxyphenol (2,6-DMP, *ε*_469_ = 27,500 M^−1^ cm^−1^), pyrogallol (*ε*_420_ = 3724 M^−1^ cm^−1^), guaiacol (*ε*_456_ = 12,100 M^−1^ cm^−1^), and hydroquinone (*ε*_247_ = 21,300 M^−1^ cm^−1^). One unit of enzymatic activity was defined as the amount of enzyme oxidizing 1 μmol of substrate per min. Kinetic measurements of laccase activity were performed for ABTS and 2,6-DMP, in 50 mM phosphate-citrate buffer pH 4. Michaelis–Menten constants were calculated by the respective nonlinear regression curves using the software GraphPad Prism 5 (GraphPad Software, Inc., U.S.A.).

### Determination of pH and temperature optima and thermal stability: biochemical characterization

The effect of pH and temperature on enzyme activity and stability was measured using ABTS (2 mM) as the substrate. The optimum pH was calculated by measuring the activity at 40 °C after incubation for 10 min over the pH range 2.0–9.0 using as buffers 0.1 M phosphate (pH 2.0), 0.1 M citrate–phosphate (pH 3.0–5.0), 0.1 M phosphate (pH 6.0–7.0), and 0.1 M Tris–HCl (pH 8.0–9.0). The optimum temperature was calculated by assaying laccase activity over the range of 20–70 °C for 10 min in 0.1 M citrate–phosphate buffer, pH 3 for *Pc*Lac1, and pH 4 for *Pc*Lac2. The thermostability was estimated after incubation of the purified enzymes at temperatures ranging from 30 to 70 °C and pH 7.0, for selected time intervals, and calculating the residual activity under standard assay conditions. The effects of various inhibitors and organic solvents on the laccase activity were determined by adding the compound at the indicated concentration in the assay mixture and by measuring the residual activity under standard assay conditions.

### Determination of formal potential

FTacV experiments [54] were performed in a single compartment three-electrode cell consisting of a disk glassy carbon electrode (GC) with a surface of 0.785 mm as a working electrode, a 1.6 mm-diameter Pt-coated titanium rod as a counter electrode, and an Ag|AgCl, KCl sat. reference electrode (+ 0.197 V vs NHE) [34]. The aqueous solution of about 3 mL consisted of 100 mM tartrate buffer (pH 4) (PENTA) as a supporting electrolyte.

Voltammetric measurements were performed by a PAR 263A Potentiostat connected to an AFG 5101 Tektronix programmable arbitrary function generator. All solutions were deaerated for at least 10 min before the experiments to avoid oxygen reduction on the electrode surface. Nitrogen gas was purged over the solution during measurement, as well. The temperature of the cell was kept steady using a thermostated bath (FALC WB-MD5). The cell temperature was recorded during the measurement.

The glassy carbon electrode was polished on a cloth with the use of 0.3 μm and 0.05 μm Al_2_O_3_. Then, washing of the electrode and sonication (5 min) was performed in distilled water. After the sonication, the electrode was washed again with distilled water. An enzyme volume of 1 μL was left on the surface of the electrode to dry for about 15 min at room temperature. The procedure was repeated once more to increase the enzyme surface concentration on the electrode. Subsequently, 1 μL of Nafion was left to dry above the deposited enzyme to complete the immobilization [55, 56]. All the formal potentials were corrected by taking into account the temperature coefficient − 1.01 mV/°C of the Ag/AgCl, KCl sat. reference electrode [57].

### Biocatalytic transformation of phenolic acids

For the laccase-mediated biotransformation of phenolic acids, 25 mg of either ferulic or sinapic acid were dissolved in 100 mM citrate–phosphate buffer (pH 4 for *Pc*Lac1 and 5 for *Pc*Lac2), containing 7.5% (v/v) DMSO. The reaction started with the addition of 1.5 Units of each enzyme to a total volume of 8 mL. Reaction mixtures were incubated in a rotary shaker at 35 °C, 200 rpm for 24 h. At selected time intervals (3, 8, and 24 h), samples were collected and were immediately frozen until further analysis.

### Analytical methods

The monitoring of the products of all enzymatic reactions was performed on an UHPLC–HRMS/MS Q-Exactive Orbitrap (Thermo) platform at negative ionization mode. The analytical methodology was optimized for the better separation of the products on a UPLC C18 (2.1 × 150 mm, 1.9 μm) reversed phased column. The gradient phase consisted of solvents A: aqueous 0.1% (v/v) formic acid and B: acetonitrile. The gradient elution was: *T* = 0 min, 5% B; *T* = 1 min, 5% B, *T* = 14 min, 95% B, *T* = 15 min, 95% B, *T* = 15.1 min, 5% B; *T* = 16 min, 5% B. The flow rate was 0.350 mL/min and the injection volume was 5 μL. The column temperature was kept at 40 °C, while the sample tray temperature was set at 4 °C. The HRMS data were acquired at a full-scan mode with mass range 120–1200 m/z. The ionization conditions were as set as follows: capillary temperature, 350 °C; spray voltage, 2.7 kV; S-lense Rf level, 50 V; sheath gas flow, 40 arb. units; aux gas flow, 5 arb. units; aux. gas heater temperature, 50 °C. For the full-scan experiments, the resolution was 70,000. The data-dependent acquisition capability has been also used at 17,500 resolution, allowing for MS/MS fragmentation of the three most intense ions of every peak exceeding the predefined threshold applying a 10 s dynamic exclusion. Normalized collision energy was set at 35%. Data acquisition and analysis have been completed employing Xcalibur (Thermo).

### Statistical analysis

All results are expressed as the mean value of three replicates. Error bars represent the standard deviation. Statistical analysis was performed with SigmaPlot v.12.5 software package (Systat Software, Inc., San Jose, CA, USA).

## Supplementary Information

**Additional file 1.** **Figure S1.** 3rd Harmonic of (a) *Pc*Lac1 and (b) *Pc*Lac2. Experimental conditions: deaerated tartrate buffer pH 4.0 100 mM, T 43^o^C, v 50 mV/s, A 180 mV and f 6 (black), 9 (red), 12 (green) and 16 (blue) Hz. **Figure S2.** Possible products from ferulic acid dimerization. Products shown were previously reported [1–4]. **Figure S3.** Possible products from ferulic acid dimerization. Products shown were previously reported [1,2,4,5].** Figure S4. **Possible products from ferulic acid trimerization. Products shown were previously reported [6–9]. **Figure S5. **Possible products from sinapic acid dimerization. Products shown were previously reported [4,5,10–12].

**Additional file 2.** Proteomic analysis of *Pc*Lac1 and *Pc*Lac2.

## Data Availability

All data supporting the conclusions of this article are included in the manuscript and its additional files. Samples of materials produced in the current work are available from the corresponding author upon reasonable request.
