# Supplementary material for: Discovery of two novel laccase-like multicopper oxidases from Pleurotus citrinopileatus and their application in phenolic oligomer synthesis
Source: Biotechnol Biofuels. 2021 Apr 1;14:83. doi: 10.1186/s13068-021-01937-7 (PMC8017616; doi:10.1186/s13068-021-01937-7)
Supplement: Supplementary file 1 — Additional file 1. Figure S1. 3rd Harmonic of (a) PcLac1 and (b) PcLac2. Experimental conditions: deaerated tartrate buffer pH 4.0 100 mM, T 43oC, v 50 mV/s, A 180 mV and f 6 (black), 9 (red), 12 (green) and 16 (blue) Hz. Figure S2. Possible products from ferulic acid dimerization. Products shown were previously reported [1–4]. Figure S3. Possible products from ferulic acid dimerization. Products shown were previously reported [1,2,4,5]. Figure S4. Possible products from ferulic acid trimerization. Products shown were previously reported [6–9]. Figure S5. Possible products from sinapic acid dimerization. Products shown were previously reported [4,5,10–12]. [file 13068_2021_1937_MOESM1_ESM.docx]

Supplementary Material for

**Discovery of two novel laccase-like multicopper oxidases from *Pleurotus citrinopileatus* and their application in phenolic oligomer synthesis**

**Zerva, A.^1^, Pentari, C.^1^, Termentzi, A.^2^, America A.H.P^3^, Zouraris, D.^4^, Bhattacharya S. K.^2^, Karantonis A.^4^, Zervakis G.I. ^5^, Topakas, E.^1*^**

^1^ Industrial Biotechnology & Biocatalysis Group, Biotechnology Laboratory, School of Chemical Engineering, National Technical University of Athens, 5 Iroon Polytechniou Str, Zografou Campus, Athens, Greece.

^2^ Department of Ophthalmology/Bascom Palmer Eye Institute, University of Miami, Miami, Florida, USA 33136

^3^ Wageningen Plant Research, Wageningen University and Research, Wageningen, The Netherlands

^4^ Laboratory of Physical Chemistry and Applied Electrochemistry, School of Chemical Engineering, National Technical University of Athens, Zografou, Athens, Greece.

^5^ Laboratory of General and Agricultural Microbiology, Agricultural University of Athens, Athens, Greece.

* Correspondence: Evangelos Topakas, vtopakas@chemeng.ntua.gr

**Table S1.** Purification of *Pc*Lac1 and *Pc*Lac2 from the culture supernatant of *P. citrinopileatus*.

|  | *Pc*Lac1 | | | *Pc*Lac2 | | |
| --- | --- | --- | --- | --- | --- | --- |
|  | U mg^-1^ | Purification (fold) | Yield (%) | U mg^-1^ | Purification (fold) | Yield (%) |
| crude | 0.47 | 1.00 | 100.00 | 0.47 | 1.00 | 100.00 |
| Q sepharose | 10.19 | 21.76 | 36.29 | 18.40 | 39.30 | 15.12 |
| DEAE | 18.68 | 39.91 | 30.37 | 27.65 | 59.07 | 11.79 |


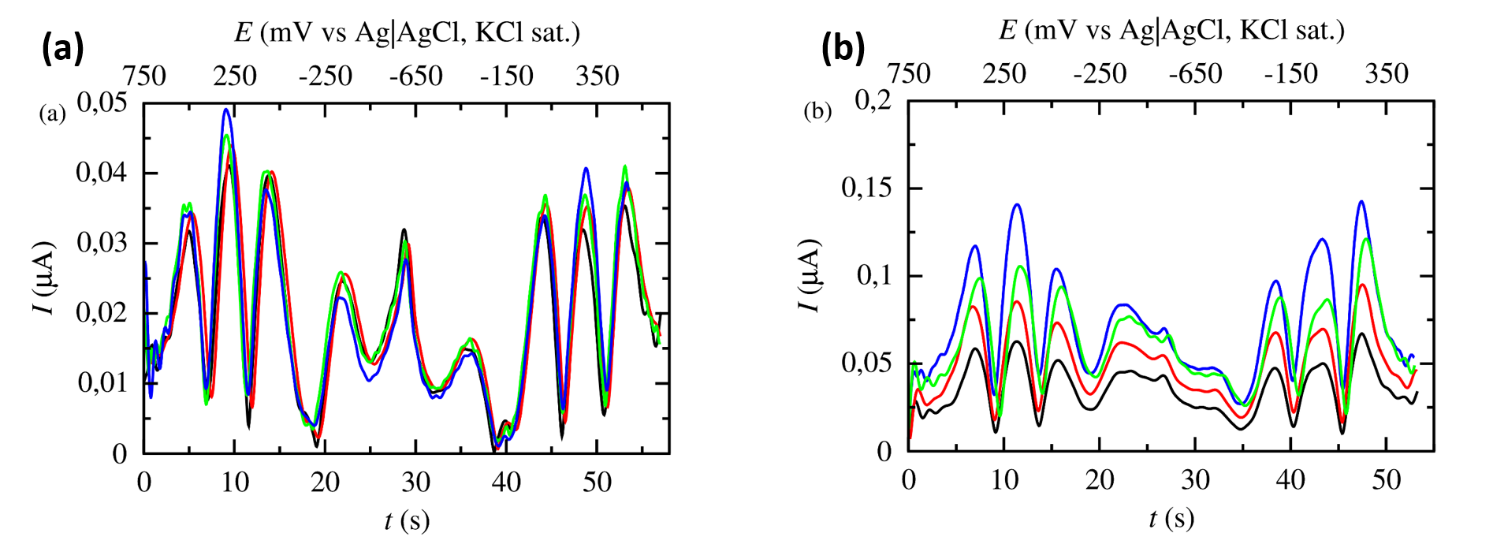


**Figure S1.** 3rd Harmonic of (a) *Pc*Lac1 and (b) *Pc*Lac2. Experimental conditions: deaerated tartrate buffer pH 4.0 100 mM, T 43^o^C, v 50 mV/s, A 180 mV and f 6 (*black*), 9 (*red*), 12 (*green*) and 16 (*blue*) Hz

**
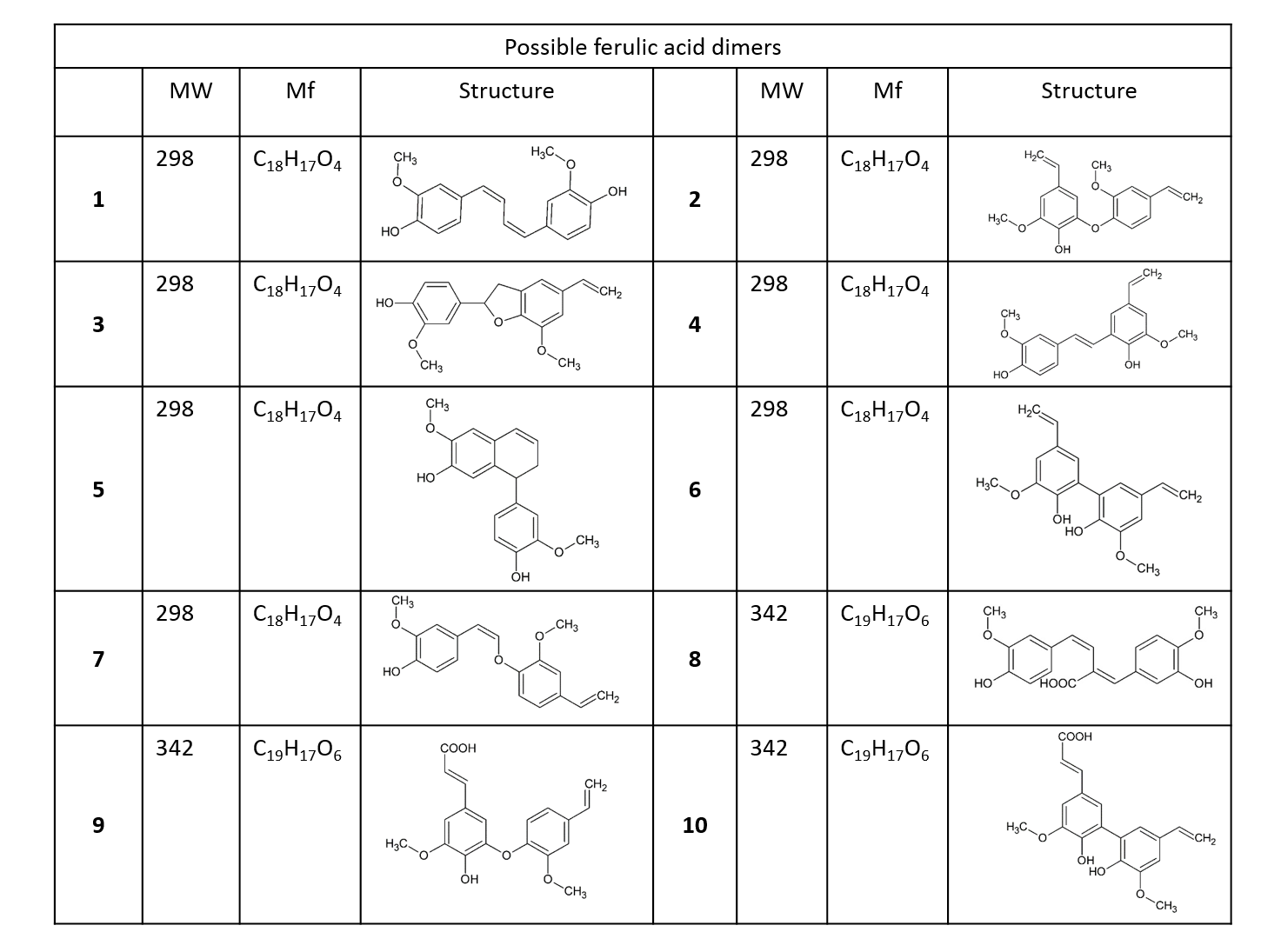
**

**Figure S2.** Possible products from ferulic acid dimerization. Products shown were previously reported [1–4].


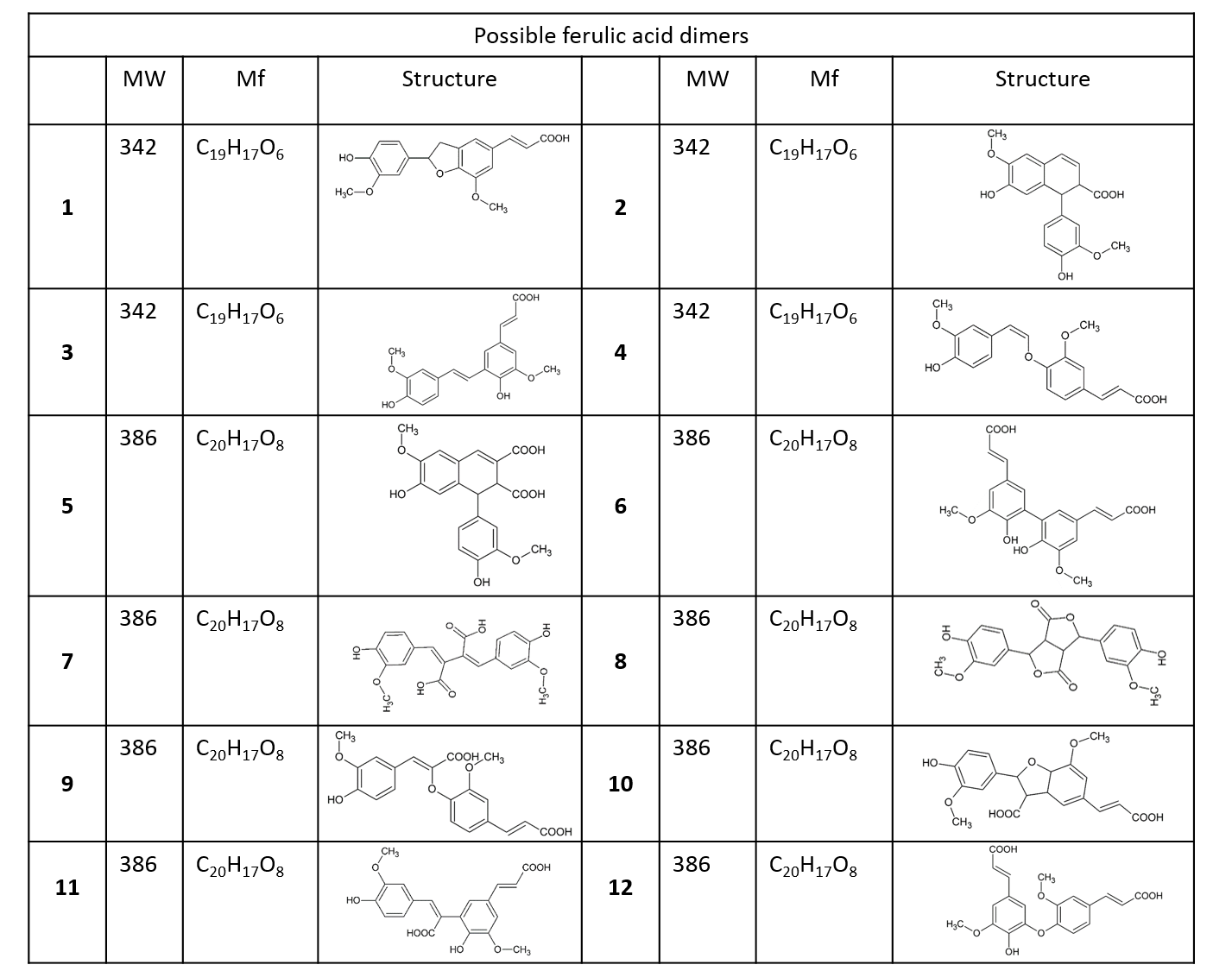


**Figure S3.** Possible products from ferulic acid dimerization. Products shown were previously reported [1,2,4,5].


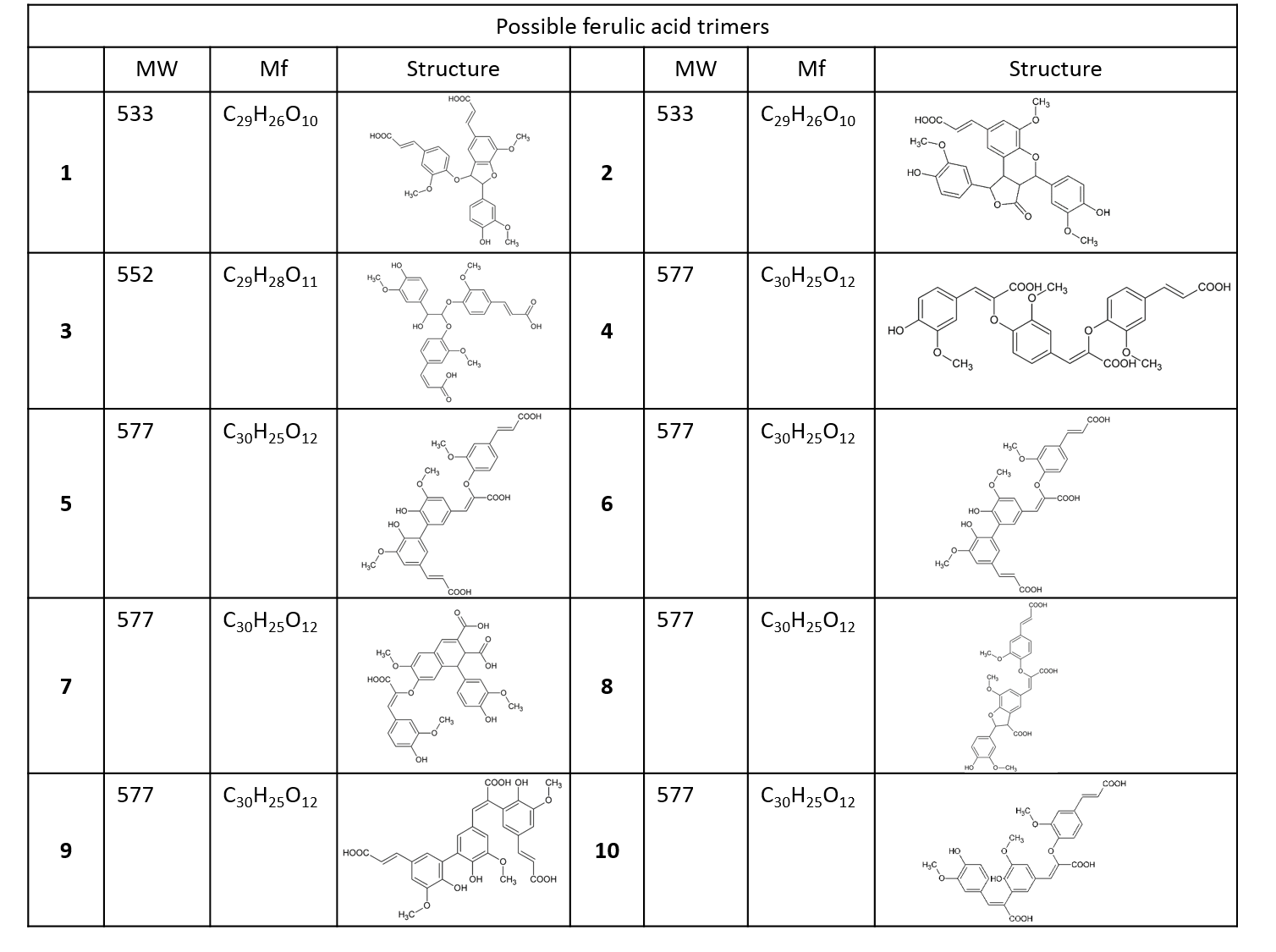


**Figure S4.** Possible products from ferulic acid trimerization. Products shown were previously reported [6–9].


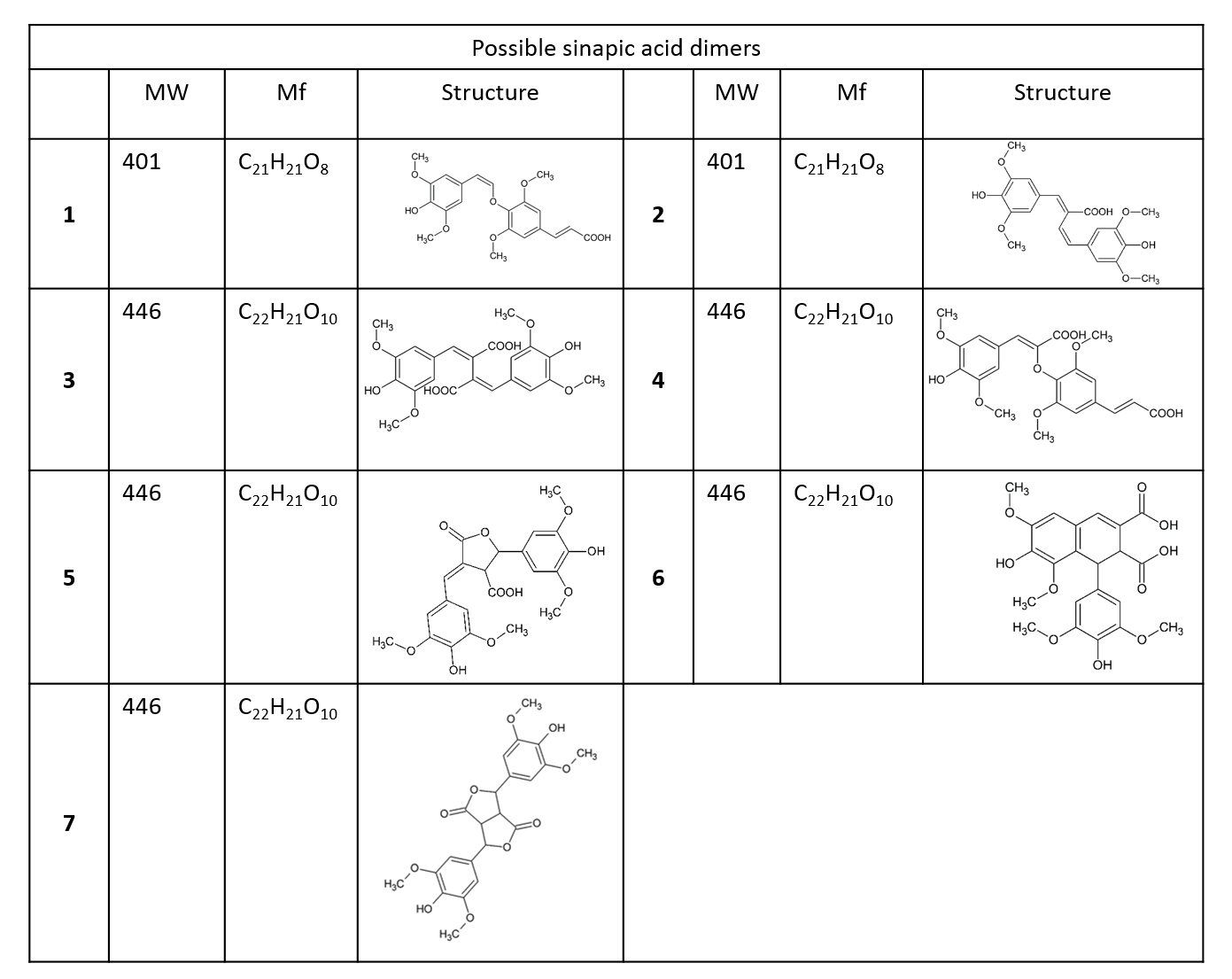


**Figure S5.** Possible products from sinapic acid dimerization. Products shown were previously reported [4,5,10–12].

**References**

1. Carunchio F. Oxidation of ferulic acid by laccase: identification of the products and inhibitory effects of some dipeptides. Talanta. 2001;55.

2. Ralph J, Lundquist K, Brunow G, Lu F, Kim H, Schatz PF, et al. Lignins: Natural polymers from oxidative coupling of 4-hydroxyphenyl- propanoids. Phytochem Rev [Internet]. 2004;3:29–60. Available from: https://doi.org/10.1023/B:PHYT.0000047809.65444.a4

3. Adelakun OE, Kudanga T, Parker A, Green IR, le Roes-Hill M, Burton SG. Laccase-catalyzed dimerization of ferulic acid amplifies antioxidant activity. J Mol Catal B Enzym [Internet]. 2012;74:29–35. Available from: http://www.sciencedirect.com/science/article/pii/S1381117711002359

4. Vismeh R, Lu F, Chundawat SPS, Humpula JF, Azarpira A, Balan V, et al. Profiling of diferulates (plant cell wall cross-linkers) using ultrahigh-performance liquid chromatography-tandem mass spectrometry. Analyst. 2013;138.

5. Perna V, Agger JW, Holck J, Meyer AS. Multiple Reaction Monitoring for quantitative laccase kinetics by LC-MS. Sci Rep. Nature Publishing Group; 2018;8.

6. Ward G, Hadar Y, Bilkis I, Konstantinovsky L, Dosoretz CG. Initial Steps of Ferulic Acid Polymerization by Lignin Peroxidase. J Biol Chem. 2001;276:18734–41.

7. Bunzel M, Ralph J, Funk C, Steinhart H. Structural elucidation of new ferulic acid-containing phenolic dimers and trimers isolated from maize bran. Tetrahedron Lett [Internet]. 2005;46:5845–50. Available from: http://www.sciencedirect.com/science/article/pii/S0040403905014218

8. Bunzel M. Chemistry and occurrence of hydroxycinnamate oligomers. Phytochem Rev [Internet]. 2010;9:47–64. Available from: https://doi.org/10.1007/s11101-009-9139-3

9. Bento-Silva A, Vaz Patto MC, do Rosário Bronze M. Relevance, structure and analysis of ferulic acid in maize cell walls. Food Chem [Internet]. 2018;246:360–78. Available from: http://www.sciencedirect.com/science/article/pii/S0308814617318071

10. Lacki K, Duvnjak Z. Transformation of 3,5-Dimethoxy,4-hydroxy Cinnamic Acid by Polyphenol Oxidase from the Fungus *Trametes versicolor*: Product Elucidation Studies. 1998.

11. Bunzel M, Ralph J, Kim H, Lu F, Ralph SA, Marita JM, et al. Sinapate dehydrodimers and sinapate-ferulate heterodimers in cereal dietary fiber. J Agric Food Chem. 2003;51:1427–34.

12. Liu HL, Wan X, Huang XF, Kong LY. Biotransformation of sinapic acid catalyzed by *Momordica charantia* Peroxidase. J Agric Food Chem. 2007;55:1003–8.
